# Severe Amitraz Poisoning: A Case of Successful Emergency Intervention

**DOI:** 10.7759/cureus.63228

**Published:** 2024-06-26

**Authors:** Waseem M Ilyas, Charuta Gadkari, Aditya Pundkar, Utkarsh Gaur

**Affiliations:** 1 Emergency Medicine, Datta Meghe Institute of Higher Education and Research, Jawaharlal Nehru Medical College, Wardha, IND; 2 Orthopaedics, Datta Meghe Institute of Higher Education and Research, Jawaharlal Nehru Medical College, Wardha, IND

**Keywords:** organophosphate, similarity, mimic, bradycardia, atropine, amitraz toxicity, amitraz poisoning

## Abstract

Amitraz poisoning is being increasingly seen in clinical practice, presenting physicians with challenges due to its rapidity of onset of severe clinical features, its similarity with organophosphate poisoning and the absence of specific antidotes. Early initiation and appropriate treatment are vital for favourable outcomes. Our case report is of a 40-year-old male who presented to us with grave clinical features following deliberate ingestion of Amitraz in a suicidal attempt. On arrival, he had bradycardia, hypotension, respiratory depression, and altered sensorium. Immediate administration of atropine stabilised his vital signs. Laboratory investigations revealed uncommon electrolyte imbalances, which were promptly corrected. The patient received supportive care in the intensive care unit (ICU), regained consciousness within three days, and was discharged after a week of hospitalisation. Despite the rapid onset and severity of symptoms caused by Amitraz poisoning, early intervention and supportive care can lead to a full recovery. This case underscores the importance of promptly recognising Amitraz poisoning and initiating treatment, its similarity with organophosphate poisoning and the role of atropine. Further research is needed to establish comprehensive management guidelines for tackling this emerging poisoning hazard.

## Introduction

Amitraz is an insecticide derived from formamidine and used as an ectoparasiticide on canines, cattle, and sheep. It is also an agricultural insecticide for fruit crops [1]. In the past few years, an increasing number of Amitraz poisoning cases have been reported. This rise may be attributed to its increased usage, partly due to the negative perception and discouragement of organophosphorus compound use [2,3]. It is sold as a 12.5% to 50% formulation in organic solvents, mostly xylene, and needs dilution in water before use. Amitraz is an α2 agonist in the central nervous system (CNS) and also acts as an α1 and α2 agonist peripherally while inhibiting the synthesis of monoamine oxidase (MAO) and prostaglandin E. It has a short duration of action, a serum elimination half-life of just four hours, and its primary end metabolite is excreted by the kidneys [1,4]. 

Human exposure to Amitraz can occur intentionally or accidentally through inhalation, skin contact, or ingestion. Its clinical presentation can resemble clonidine poisoning due to its α2 adrenergic agonist action, as well as organophosphate/carbamate poisoning [1,4,5]. The United States Environmental Protection Agency (EPA) has described it under Group C carcinogens, suggesting possible carcinogenicity and also as a moderately toxic compound via the dermal route, and as a slightly toxic compound via oral and inhalation route for humans [4,5]. Healthcare workers appear to have an inadequate awareness of the toxicity and treatment of Amitraz, which, coupled with mostly uneventful recoveries in reported cases, may contribute to the underreporting of Amitraz poisoning incidents. To date, only a few deaths have been documented in the medical literature attributed to Amitraz poisoning [4-6]. In this case report, we detail the successful treatment of a severe Amitraz poisoning case upon arrival.

## Case presentation

A forty-year-old male was admitted to the emergency department after ingesting pesticide along with alcohol four hours earlier with suicidal intent. On arrival, he was found unconscious with a Glasgow Coma Scale (GCS) score of three (E1V1M1). His heart rate was 34 beats per minute, with the cardiac monitor displaying sinus bradycardia, and his blood pressure measured 80/50 mm Hg. The respiratory rate was 18 breaths per minute, and oxygen saturation was 68% in room air, improving to 96% with 12 liters of oxygen supplementation. Bilateral basal crepitations were noted on auscultation. His bedside glucose was 119 mg/dL, and pupils were normal in size and equally reactive to light.

Following the 2020 Advanced Cardiovascular Life Support (ACLS) protocol for bradycardia, the patient received an immediate 1 mg dose of atropine, resulting in a heart rate increase to 120 beats per minute and a rise in blood pressure to 90/60 mm Hg. Subsequently, the patient was intubated, sedated, and underwent gastric lavage. The pesticide responsible for the poisoning was identified as “Amitraz” by the patient’s relatives. As the compound was unfamiliar, quick online research was conducted to ascertain treatment options, which revealed supportive care as the mainstay based on a few case reports.

Routine laboratory investigations included complete blood counts, liver and renal function tests, arterial blood gas analysis, and coagulation profile. The patient exhibited mild electrolyte imbalances, with hypomagnesemia (serum magnesium level: 1.4 mg/dL) and low calcium levels (7.1 mg/dL), which were corrected by magnesium sulphate and calcium gluconate. Cholinesterase levels and the rest of the blood investigations were within normal range (Table 1). A computerised tomography (CT) brain scan revealed no abnormalities. The patient received conservative management in the intensive care unit (ICU), including antibiotics, antacids, thiamine, and multivitamins. After three days, he regained consciousness and maintained spontaneous respiration, leading to successful extubation. Following an additional four days of observation and treatment, the patient was discharged with complete recovery.

**Table 1:** Blood investigation report of the patient

Laboratory investigations	Patient’s value	Normal value
Haemoglobin	13.9 gm%	14- 18 gm%
WBC count	10,800 /cu.mm	4000- 11,000 /cu.mm
Platelet count	259,000 /cu.mm	150,000 to 450,000 /cu.mm
Haematocrit	42.9%	41% to 50%
Renal function test		
Urea	17 mg/dl	15 - 36 mg/dl
Creatinine	0.7 mg /dl	0.5 - 1.3 mg /dl
Sodium	143 mmol/L	135 - 145 mmol/L
Potassium	4.9 mmol /L	3.5 - 5.1 mmol /L
Other investigations		
Calcium	7.1 mg/dL	8.4 - 10.2 mg/dL
Magnesium	1.4 mg/dL	1.6 - 2.3 mg/dL
Phosphorus	4.0 mg/dL	2.5 - 4.5 mg/dL
Serum cholinesterase	6.4 kU/L	5.3 - 12.9 kU/L

## Discussion

Most cases of Amitraz poisoning (56.5%) are reported in children [4]. The most common route of exposure is ingestion, followed by dermal exposure. Poisoning typically occurs accidentally, though suicidal ingestion is also noted. Intentional percutaneous exposure in humans has occurred in some areas where Amitraz is used in the treatment of scabies. There has been one reported case of homicidal poisoning [4]. Symptoms appear earlier, and recovery is delayed with oral ingestion compared to percutaneous exposure. The lethal dose of Amitraz is not precisely known, but some studies suggest a dose of 200 mg/kg may be lethal. For an average adult weighing 60 kg, this corresponds to a potentially lethal dose of 12 grams [4,5,7].

The most common clinical features of Amitraz poisoning include bradycardia, altered sensorium, constricted pupils, vomiting, elevated blood glucose, and respiratory failure, which are seen in more than one-third of patients. Less common symptoms, seen in less than one-third of patients, include low blood pressure, seizures, hypothermia, polyuria, dilatation of pupils, elevated transaminases, and aspiration pneumonia. Other rarely seen clinical manifestations are incoordination in arms and legs, hallucinations, reduced motor tone, cerebral oedema, raised blood pressure, tachyarrhythmias, fever, low blood glucose levels and gastrointestinal manifestations like dry mouth, reduced intestinal motility, distended abdomen, and Ogilvie’s syndrome. Amitraz can also cause laboratory abnormalities such as transaminitis, dysglycemia, and dyselectrolytemia, with hyponatremia reported in a few cases [4,5].

The toxicity of Amitraz can also be partly attributed to its solvent, xylene. In one case report, a patient with Amitraz poisoning had severe hemolytic anaemia necessitating transfusions and acute kidney injury (AKI) requiring hemodialysis, which have also been noted in xylene poisoning literature. Xylene toxicity can also lead to clinical features like altered sensorium, ataxia, and nystagmus [4,5].

In our patient, the clinical presentation included altered sensorium and bilateral lung crepitations likely due to pulmonary oedema, respiratory depression, bradycardia, and hypotension. These signs initially suggested organophosphate poisoning, which is more commonly associated with such features. This case highlights how Amitraz poisoning can closely resemble organophosphate poisoning. Therefore, doctors must recognize this similarity to ensure accurate diagnosis and appropriate treatment [3,5].

The treatment of Amitraz poisoning is essentially symptomatic, as no specific antidote has been found. Treatment involves stabilizing hemodynamics through adequate hydration, maintaining airway patency, administering oxygen, minimizing toxin absorption, and enhancing toxin elimination from the body. Early gastric lavage can prevent severe complications, while the clinical benefits of activated charcoal remain uncertain. Experimental studies on animals have shown success with α2 adrenergic antagonist drugs like yohimbine and atipamezole. In one case, yohimbine led to rapid neurological recovery within 24 hours. However, evidence supporting their use as antidotes for Amitraz poisoning in humans is lacking in the literature. Despite the severe clinical features, Amitraz poisoning has low mortality and a good prognosis [4,5,8].

Symptoms typically emerge within 30 to 180 minutes post-exposure, with most studies indicating onset within three hours. However, reports suggest a broader range of five minutes to six hours for oral ingestion and up to 24 hours for dermal exposure, underscoring the potency of Amitraz. In our patient’s case, CNS depression developed rapidly, with the lowest GCS score at presentation within four hours, likely exacerbated by alcohol consumption. The duration of CNS depression is proportional to the volume ingested, and most of the patients regain consciousness within two days, probably due to the short half-life of the compound. Persistent altered sensorium beyond this period warrants investigation into alternative causes. Cerebral oedema was documented in brain imaging studies in two cases, while seizures occurred in less than one-third of the cases [4,5].

Our patient presented with severe bradycardia, with a heart rate as low as 34 beats per minute and a blood pressure of 80/50 mm Hg. Sinus bradycardia was evident on cardiac monitoring. Administering one dose of 1mg atropine improved heart rate and blood pressure and obviated the need for further atropine during the treatment period. The circulatory system remained stable with supportive care alone thereafter. While the use of atropine in Amitraz poisoning is controversial in medical literature, it has shown efficacy in improving bradycardia in many cases. In some instances, dopamine has been utilised for bradycardia treatment instead. Some experts have suggested that atropine be only used for those patients with symptomatic bradycardia and unnecessary in cases with asymptomatic bradycardia or constricted pupils [1, 4-6]. Nevertheless, in our patient, prompt administration of atropine likely played a lifesaving role, as any delay in hospitalisation could have resulted in death from circulatory shock.

Our patient also had dyselectrolytemia, characterised by hypocalcemia and hypomagnesemia in the initial period. These were corrected by calcium gluconate and magnesium sulphate, following which the electrolyte levels remained within normal range throughout the rest of the treatment period. A delayed arrival at the hospital could have resulted in a severe presentation of these abnormalities, complicating treatment. Hence, clinicians must remain vigilant regarding these potential complications when managing cases of Amitraz poisoning [4-6].

Our patient experienced altered sensorium and circulatory shock within just four hours. The short half-life of Amitraz was probably the reason for the absence of return of bradycardia, electrolyte imbalances, and the relatively brief duration of unconsciousness. The prompt initiation of atropine and aggressive treatment saved the patient and led to full recovery, which suggests that despite its rapid onset and severe effects, the chemical’s effect is relatively short-lived, and early intervention can lead to positive outcomes [7]. Thus, Amitraz users and healthcare workers must recognise its toxic potential and prioritise timely healthcare provision.

## Conclusions

Amitraz poisoning manifests rapidly with severe clinical features and can be fatal without prompt treatment. However, mortality is generally low due to its short half-life and good treatment response. While atropine has been found beneficial for bradycardia in many case reports of Amitraz poisoning, its use is still debated; however, our study found it lifesaving. The electrolyte imbalances, such as hypocalcemia and hypomagnesemia, found in our patient are uncommon in Amitraz poisoning and could complicate delayed treatment, underscoring the need for vigilant monitoring. In cases of unknown compound poisoning, clinicians should consider Amitraz as a potential cause alongside organophosphate poisoning to prevent unnecessary or excessive treatment. Further research is needed to establish clear treatment guidelines, and increased awareness among healthcare workers is essential due to the rising incidence of Amitraz poisoning cases.
